# Occipital nerve stimulation for the treatment of intractable chronic migraine headache: ONSTIM feasibility study

**DOI:** 10.1177/0333102410381142

**Published:** 2011-02

**Authors:** Joel R Saper, David W Dodick, Stephen D Silberstein, Sally McCarville, Mark Sun, Peter J Goadsby

**Affiliations:** 1 Michigan Head–Pain and Neurological Institute, USA.; 2 Mayo Clinic, USA.; 3 Jefferson Headache Center at Thomas Jefferson University, USA.; 4 Medtronic Neuromodulation, USA.; 5 University of California, San Francisco, USA.

**Keywords:** Intractable chronic migraine headache, chronic migraine headache, device implantation, headache days, occipital nerve stimulation

## Abstract

*Background:* Medically intractable chronic migraine (CM) is a disabling illness characterized by headache ≥15 days per month.

*Methods:* A multicenter, randomized, blinded, controlled feasibility study was conducted to obtain preliminary safety and efficacy data on occipital nerve stimulation (ONS) in CM. Eligible subjects received an occipital nerve block, and responders were randomized to adjustable stimulation (AS), preset stimulation (PS) or medical management (MM) groups.

*Results:* Seventy-five of 110 subjects were assigned to a treatment group; complete diary data were available for 66. A responder was defined as a subject who achieved a 50% or greater reduction in number of headache days per month or a three-point or greater reduction in average overall pain intensity compared with baseline. Three-month responder rates were 39% for AS, 6% for PS and 0% for MM. No unanticipated adverse device events occurred. Lead migration occurred in 12 of 51 (24%) subjects.

*Conclusion:* The results of this feasibility study offer promise and should prompt further controlled studies of ONS in CM.

## Introduction

Migraine is ranked by the World Health Organization as among the world’s most disabling medical conditions (1), affecting 12% of the US population: 18% of women and 6% of men (2). During the course of their illness, which often begins in childhood or adolescence, approximately 3% to 14% of migraine patients will progress to chronic migraine (CM), with more than half of the days of each month in pain (3). Despite major advances in understanding the pathogenesis of migraine, new pharmacologic treatments (4) and the availability of intensive systems of care for difficult cases, in many patients migraines remain intractable to medical therapy (5).

In 1999 Weiner and Reed reported the beneficial effects of subcutaneous occipital nerve stimulation (ONS) in 12 of 13 patients who they believed to have occipital neuralgia (6). Leads were placed in the subcutaneous tissue superficial to the cervical musculature and fascia transversing the occipital nerves at the level of C1. A review of Weiner’s cases by author JRS at the behest of Medtronic Neuromodulation resulted in a challenge to the diagnosis of occipital neuralgia (unpublished results). JRS subsequently recommended that these patients be evaluated by author PJG using functional neuroimaging (positron emission tomography). These studies, performed in eight patients, demonstrated brain changes (phenotype and imaging signature) consistent with CM (7).

Published reports from open-label studies have demonstrated possible efficacy of ONS in a variety of primary headache disorders, including CM (8,9), cluster headache (10), occipital neuralgia (11) and hemicrania continua (12). These findings, those of Weiner and Reed (6) and those of Goadsby and colleagues (7) prompted the development of a feasibility study. The goals of the trial were to determine whether a well-designed, controlled study that included a valid placebo arm could demonstrate insights into the potential benefits and risks of this new therapy. Among the potential risks to be assessed were lead migration, lead fracture, skin erosion, infection, loss of effect, muscle spasm and battery malfunction or depletion. Preliminary results have been presented (American Headache Society Annual Scientific Meeting, Boston, MA, June 26, 2008; European Headache and Migraine Trust International Congress, September 2008; and American Academy of Neurology, Seattle, WA, April 2009) (13,14).

## Methods

The study was prospective, multicenter, randomized, blinded, and placebo-controlled. It was designed to obtain preliminary safety and efficacy data for ONS treatment of CM. As a feasibility study, especially in a patient population that has been the focus of very few randomized controlled trials, no primary endpoint was prespecified; rather, a range of efficacy measures was identified and evaluated at three months in comparison to baseline. Among the endpoints measured were reduction in headache days per month, decrease in overall pain intensity (0–10 scale) and responder rate (i.e. percentage of patients with a ≥50% drop in headache days per month or a ≥3-point drop in overall pain intensity from baseline, based on daily electronic diary data). A headache day was defined as each day that a subject rated his or her overall headache pain intensity as ≥3. CM was diagnosed using the second edition of the International Classification of Headache Disorders (ICHD-II) (15). Subject enrollment criteria included (i) headaches occurring on 15 or more days per month for more than three months in the absence of medication overuse, (ii) pain involving the occipital or suboccipital region and (iii) pain refractory to preventive medications. Key inclusion and exclusion criteria are listed in Table 1.

**Table 1.** Inclusion and exclusion criteria

Inclusion	• Diagnosis of CM headache as defined by the 2004 IHS criteria:
	◦ Migraine headache occurring on 15 or more days/month for more than three months in absence of medication overuse.
	◦ Not attributed to another disorder.
	• Headache pain defined by the following criteria:
	◦ During each of two consecutive periods of four consecutive weeks, a minimum of 15 days of CM headache with peak pain intensity ≥5 (on a 0–10 scale).
	◦ Subject may have headache of any intensity (0–10 scale) on days over 15 during each four-week period.
	◦ Headache pattern has been present for 12 months or longer.
	◦ Refractory, as determined by failure to respond or intolerance to an adequate trial of preventative medications from at least two different classes of drugs.
	• Headache is characterized by:
	◦ Pain located between C3 level to vertex.
	◦ Any location between ears (i.e. occipital or suboccipital region within distribution of greater and/or lesser occipital nerves).
	◦ Pain may be unilateral or bilateral and may include pain in frontal, temporal or retro-orbital region or into neck/shoulder location.
	• Onset of migraine headache occurred before age 50 years.
	• Current acute and prophylactic headache medication regimens have been stabilized for four weeks prior to preliminary enrollment visit.
	• Response to a temporary, short-acting anesthetic block to the occipital distribution was positive.
	• Subject is age 18 years or older and has signed informed consent form.
	• Subject will be available for appropriate follow-up for the duration of study and is willing and able to maintain current medication regimens during enrollment process and through three-month follow-up visit.
	• In physician’s opinion, subject is willing and able to use electronic daily questionnaire equipment.
	• Female subject of childbearing potential has negative pregnancy test at confirmation of enrollment visit, is not nursing and agrees to use adequate birth control methods for duration of study.
Exclusion	• In physician’s opinion subject has health conditions or concerns that would render them unable to participate, would impact ability of subject to adequately assess incremental effects of ONS treatment, could possibly be aggravated by treatment or confound ability to interpret results (including, but not limited to, intractable epilepsy, active major depression, psychosis, somatoform disorder, severe personality disorder). Other conditions to be considered include cardiac arrhythmias, cognitive impairment and peripheral neuropathy.
	• Previous destructive ganglionectomy, rhizotomy section or neurectomy procedure affecting C2/C3/occipital distribution.
	• Subject is not candidate for or is not willing to undergo surgical implantation of neurostimulator system.
	• Subject is deemed by investigator to have rebound headaches, and/or subject reports regular use on three or more days per week of acute medication that can cause rebound headaches.
	• Subject has participated in:
	◦ Three clinical trials for headache, in last five years or
	◦ Previously terminated from this clinical trial or
	◦ Another neurological device or drug trial within last 90 days.
	• Subject has other implanted electrical stimulation device(s) or any metallic implant or is expected to require an implant, including:
	◦ Cardiac demand pacemakers or defibrillators
	◦ Cochlear implant
	◦ CSF shunt
	◦ Aneurysm clip
	◦ Spinal cord stimulator
	• Neurostimulation (implanted or external) for headache or other head or neck pain was received within last year.
	• Significant psychological signs on examination and/or history, or has serious drug habituation or behavioral problems that in physician’s judgment renders that person inappropriate for study.
	• Unresolved legal issues related to their pain that is being assessed in this study.
	• Failure to complete at least 23 out of 28 days, during two consecutive 28-day periods, of electronic daily questionnaire during enrollment process.
	• Alternative therapy to treat headache pain (e.g. massage, biofeedback, bracing) is being used or will be used.
	• MRI or diathermy may be required.
	• Other medical or neurological conditions that would confound study.

CM = chronic migraine. IHS = International Headache Society. ONS = occipital nerve stimulation. CSF = cerebrospinal fluid. MRI = magnetic resonance imaging.

### Study groups

**Figure 1.**
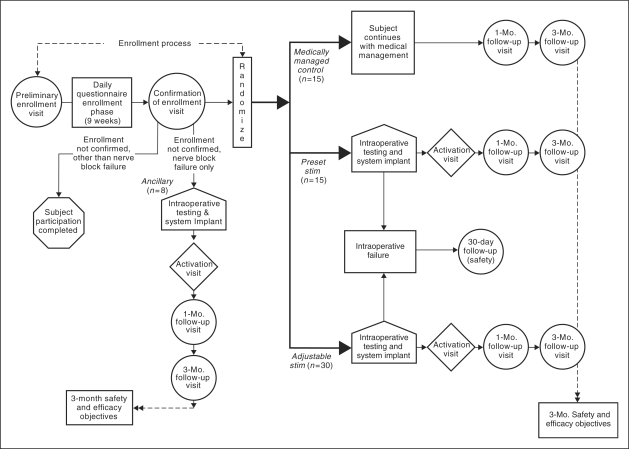
Randomization and study design, three-month overview. Adjustable stim = adjustable stimulation group. Preset stim = preset stimulation group.

Subjects who met enrollment criteria were then randomized into one of three treatment groups, adjustable stimulation (AS), preset stimulation (PS) and medical management (MM), using a randomization ratio of 2:1:1, respectively. After implantation, the AS group was instructed to maintain the stimulator in the “on” position and to adjust the device to minimize pain. Serving as the control group for the AS group were subjects who received an implanted device that provided PS rather than AS. For these patients the device was set at a stimulation setting for one minute each day during the blinded phase of the study. A third group, also serving as a control group, received only MM during the blinded phase of the study. Unlike subjects in the AS and PS groups, who were required to maintain stable medication regimens (although frequency and dose of acute medications could change if necessary), subjects in the MM group were able to adjust, change and optimize medication regimens as directed by their physicians. A fourth group, the ancillary group, met all entry criteria except response to occipital nerve block (ONB), which was an entry criterion for the other groups. A lack of response to ONB was defined as a failure to experience at least a 50% reduction in migraine pain within 24 hours of the injection of 3–5 ml of 0.5% bupivacaine into each greater occipital nerve distribution. Patients in the ancillary group were implanted and allowed to adjust the stimulation and were treated identically to the AS group. Figure 1 illustrates the randomization and study design scheme.

### Sites and blinding

The evaluation was conducted at seven centers in the US, one center in Canada, and one in the UK. The distributions of enrollment and treatment assignments by investigational center are shown in Table 2. A neurologist (headache specialist) was first identified at each center as the principal investigator except at two centers, where an implanter, usually an anesthesiologist, was first identified as the principal investigator. All headache specialists were blinded to the subjects’ group assignments and were responsible for establishing the diagnosis, optimizing subjects’ medications and evaluating subjects’ headaches at follow-up visits. None of the implanters were blinded to the subjects’ group assignments, and all were responsible for follow-up with subjects on device implantation, device activation and programming.

**Table 2.** Distribution of enrollment and subjects analyzed by investigational center

Investigational center	Subjects enrolled	Assigned to treatment group	In 3-month analysis
UK — National Hospital for Neurology and Neurosurgery	18	12	12
US — North County Neurology Associates	17	11	11
Canada – Foothills Medical Centre	13	10	10
US — Jefferson Headache Center at Thomas Jefferson University	13	10	6
US — Mayo Clinic (Scottsdale)	11	9	9
US — Henry Ford Hospital	11	7	7
US — Oklahoma University Physicians – Pain Medicine	11	6	2
US — Michigan Head-Pain and Neurological Institute	9	7	6
US — University of Colorado Health Sciences Center	7	3	3
Total	110	75	66

After confirmation of eligibility by the headache specialists, subjects were randomized into three treatment groups. Randomization was balanced across all centers but not within each center due to the anticipated relatively small number of subjects per center. The randomization was not stratified for baseline characteristics. A central randomization process provided and managed by Medtronic Neuromodulation (Medtronic) assigned a unique randomization code to each subject. Initially, randomization revealed only whether a subject was assigned to “medically managed” or “device implanted.” To maintain blinding in the device-implanted group, a sealed envelope with the complete randomization assignment (level of stimulation) was sent to implanter site personnel by Medtronic to be opened at the activation visit. Subjects were blinded to the anticipated value of adjustable stimulation over that of the preset stimulation. The sponsor’s study personnel (Medtronic) were not blinded to the randomized treatment assignments for individual subjects.

### Device

The study was conducted using the Medtronic model 7427 Synergy and model 7427 V Synergy Versitrel implantable pulse generators, model 3487A Pisces Quad and model 3887 Pisces Quad-Compact leads, model 7489 and model 7482 extensions, model 3550-25 accessory kit, model 3655-60 tunneling tool kit, model 8840 clinician programmer, model 8870 application card, model 3628 dual-screen test stimulator and model 7435 Synergy EZ patient programmer. The product specifications of stimulation parameters for both models of implantable pulse generator are the same and as follows:

- Pulse amplitude: 0–10.5 V
- Pulse rate: 3–130 Hz
- Pulse width: 60–450 µs

### Study procedures

#### Entry to study.

After approval of regulatory agencies, institutional review boards or ethics committees, subjects who provided written informed consent and met eligibility criteria were enrolled to the study. The enrollment process had three steps: preliminary enrollment visit, daily questionnaire enrollment phase and confirmation of enrollment visit. At the preliminary enrollment visit, information was collected, including health and well-being status, medical history and medication history. A preliminary diagnosis was also rendered. During the daily questionnaire enrollment phase, subjects completed an electronic daily questionnaire (EDQ) for a minimum of nine weeks, providing information about their headache, daily functional ability and medications taken. During the first week, subjects familiarized themselves with the questionnaire equipment. The purpose of the last eight weeks before the confirmation of enrollment visit was to establish baseline headache data. During the confirmation of enrollment visit, data from the EDQ was evaluated for diary compliance and headache features. Subjects who continued to meet eligibility requirements were given a short-acting ONB by the neurologist investigator. Those with a positive response to the ONB were eligible for randomization to the study. The first eight subjects study-wide who did not respond to ONB were entered into the ancillary group*.* Subjects who did not complete the enrollment process, did not respond to ONB (after the ancillary group was filled) or who did not wish to continue had no further participation in the study.

#### Implantation.

Using local anesthesia and fluoroscopic guidance, one or two leads were implanted subcutaneously, superficial to the fascia and muscle layer at the level of C1. Intraoperative testing was consistently performed according to the ONS *System Manual* to determine if a subject’s response to stimulation, as judged by adequate paresthesia coverage of the area of headache pain, was appropriate to receive a full implant. There was no trial of stimulation treatment. If during intraoperative testing the implanter believed inadequate paresthesia occurred over the location of pain based on the patient’s responses, the leads were removed, and the subject was followed for 30 days for safety and then terminated from the study. Subjects who felt adequate paresthesia over the target pain location during intraoperative testing continued with the implant procedure. Final lead placement was identified by X-ray. The implant procedure was performed with additional intravenous sedation to reduce patient discomfort; after lead placement was determined, the lead was locked in place using the twist connector, and a winged anchor was sutured in place. Connectors and extensions were used to allow placement of the neurostimulator just under the skin in the abdomen to reduce lead migration; if the abdominal site was determined to be inappropriate by the physician, the buttock was used. To further reduce the incidence of lead migration, the lead extension was placed with circular coils, creating strain-relief loops. Not all of these implant techniques, such as use of strain-relief loop, were employed consistently at the beginning of or during the study. However, a recommendation of using strain-relief loop and preference of abdominal to buttock implant location of the neurostimulator was provided to all implanters during the study when a number of lead migrations were reported.

The device was activated after the surgical site healed, between 7 and 14 days after implantation. All subjects received ONS using parameters optimized by the physician on the basis of their response to treatment, but the duration of stimulation differed according to group assignment. Patients in the PS group received one minute per day of stimulation and were instructed that their neurostimulator had been pre-programmed to deliver the correct amount of stimulation as determined by their treating physician. Patients in both the AS and PS groups were not informed of the predicted effectiveness of their treatment. Patients in the AS and ancillary groups received a device programmer allowing them to turn the neurostimulator on and off and to make minor adjustments to settings, and they were instructed to maintain the stimulator in the “on” position as much as possible. Subjects in the PS group were not given a device programmer to adjust the settings.

#### Follow-up visits.

All enrolled subjects had follow-up visits at one and three months. Safety and efficacy were evaluated at the three-month visit. Subjects in the PS and MM (control) groups were offered adjustable ONS therapy after the three-month follow-up visit. Subjects in the ancillary group were treated in the same manner as AS subjects and followed the same visit schedule. Subjects in the AS, PS and AG groups who were on medications were required to maintain stable medicine regimens, although the frequency and dose of acute medications could change if necessary. Those in the MM group were able to adjust and change medication regimens throughout the three-month blinded phase, as directed by their physicians. After the three-month follow-up visit, all subjects, regardless of initial group, were able to adjust acute and prophylactic medication regimens as needed.

The ONSTIM feasibility study has completed three-month blinded follow-up visits for safety and efficacy endpoints. Subsequent open-label, long-term follow-up visits included 6, 12, 24, and 36 months. Not all of these long-term follow-up visits have been completed at the time of this manuscript; hence, only three-month safety and efficacy results are reported here.

### Safety

The three-month safety objectives were evaluated by determining adverse device-related events (ADEs) for all implanted subjects, including ADEs reported during intraoperative testing and scheduled and unscheduled visits throughout the three-month follow-up period. ADEs were classified into those related to the components of the system, the implant procedure, device programming or device stimulation.

Non–device-related adverse events also were collected for all subjects at scheduled and unscheduled visits beginning with the confirmation of enrollment visit and throughout the three-month follow-up period. They were classified using the MedDRA Version 8.0 dictionary and the Medtronic Neuromodulation *Device Event Dictionary*.

Per protocol, there was no data safety monitoring board (DSMB) or clinical events committee (CEC) for this feasibility study. All adverse events regardless of device relatedness were reviewed and monitored by the Medtronic Neuromodulation medical advisor throughout the study. A serious unanticipated ADE or a percentage of subjects experiencing a specific serious ADE higher than previously reported were defined as criteria for consideration of modification or termination of the study.

### Data collection

Data were collected using electronic diary for headache days, pain and duration measurements. Paper case report forms were used for data collection of safety and quality of life. Data collected for safety objectives included device-related and non–device-related adverse events; data collected for efficacy objectives included headache days, headache-free days, days with prolonged and severe headache, headache pain intensity, headache duration, responder to ONS therapy, functional impairment (functional disability scale), migraine disability assessment (MIDAS), quality of life (SF-36) and subject satisfaction.

### Analytic considerations

The sample size was chosen to gain experience with ONS therapy for the treatment of CM. In order to evaluate effectively the study design, a sample size of 24 subjects in the AS group and 12 subjects in each of the PS and MM groups was required. In keeping with the exploratory nature of the study, it was not powered for a single primary endpoint. However, according to the protocol, statistical analysis was performed to allow more critical consideration of the data in order to identify factors and nuances that might be helpful in further studies. A per-protocol analysis, including all subjects who completed the electronic diary during the three-month blinded follow-up, was used to compare subjects who completed three months of stimulation therapy in the AS group with subjects in the PS, MM and ancillary groups who also completed three months of follow-up. Pairwise comparisons between the AS group and each of the three other groups were not adjusted for multiple comparisons and are presented only as a guide to interpreting the study. We considered differences with *p* < .05 as potentially informative, and these are nominally referred to as statistically significant throughout the paper, with actual *p* values not reported due to the exploratory nature of the analyses. Wilcoxon’s rank sum tests were used to analyze headache days, pain intensity, disability and quality-of-life outcomes; these summary data are presented as mean ± standard deviation. Fisher’s exact tests were used to analyze responder rate and subject satisfaction; these summary data are presented as frequency counts and percentages. For the safety objective of the study, descriptive summaries are presented. SAS software (version 9.1, SAS Institute, Cary, NC, USA) was used for all data analyses.

An interim analysis was conducted in January 2007 for business planning of future studies. No stopping rules were applied because the analysis was unrelated to safety, which is monitored and evaluated on an ongoing basis as described above. The results of this interim analysis were not provided to the investigators.

## Results

**Figure 2.**
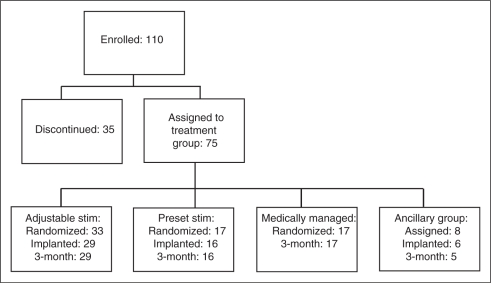
Disposition of patients in the study. Adjustable stim = adjustable stimulation group. Preset stim = preset stimulation group.

The clinical investigation began on May 26, 2004, and the last subject was enrolled on April 6, 2007. The final three-month follow-up visit was conducted on November 13, 2007. A total of 110 subjects aged 18 years and older were enrolled to achieve the final sample size of 75 subjects assigned to a treatment group (Figure 2). Thirty-five subjects discontinued the study prior to being assigned to a treatment group. The most common reasons for discontinuation were failure to meet the confirmation of enrollment criteria, withdrawal of consent or physician-determined withdrawal prior to treatment assignment. After meeting the study inclusion criteria, subjects were randomized into the study groups: 33 subjects in the AS group; 17 subjects in the PS group; and 17 subjects in the MM control group (ratio 2:1:1, respectively). Eight subjects were entered into the ancillary group to evaluate the predictive value of ONB. Of the 75 subjects assigned to a treatment group, eight discontinued prior to the end of the three-month blinded phase of the study: four subjects withdrew consent prior to implant (two AS, one PS, 1 ancillary group); two subjects were intraoperative failures (one AS, one ancillary group); one AS subject was lost to follow-up prior to implant; one ancillary group subject discontinued after the one-month follow-up visit because of lack of efficacy. Of the 67 subjects who continued to the three-month blinded follow-up, one subject (AS) did not complete the EDQ between implant and three months; 66 subjects (28 AS, 16 PS, 17 MM, 5 ancillary group) completed the EDQ through the three-month follow-up period.

The subjects experienced migraine for an average of 22.0 years prior to the study (range, 1–51 years) and experienced CM headaches for an average of 10.0 years prior to study enrollment (range, <1–30 years). The gender ratio of males to females was approximately 1:4. Treatment groups were similar in demographic and baseline headache characteristics (Table 3).

**Table 3.** Patient demographics and characteristics

	Treatment group				
Patient baseline characteristics	Adjustable stimulation* (*N* = 28)	Preset stimulation (*N* = 16)	Medically managed (control) (*N* = 17)	Ancillary group (*N* = 5)	Total (*N* = 66)
Age (years, mean ± SD)	41 ± 11.6	44 ± 10.0	44 ± 10.2	50 ± 6.4	43 ± 10.6
Gender ratio (F/M)	22/6 79%/21%	13/3 81%/19%	15/2 88%/12%	3/2 60%/40%	53/13 80%/20%
Headache history					
Duration of migraine (years migraine experienced prior to study entrance, mean ± SD)	21 ± 12.4	22 ± 9.8	25 ± 13.7	18 ± 15.1	22 ± 12.3
Disability scores (mean ± SD)	4.0 ± 0.2	3.9 ± 0.3	4.0 ± 0.0	4.0 ± 0.0	4.0 ± 0.2
Number of headache days per month (mean ± SD)	22.4 ± 6.3	23.4 ± 5.1	23.7 ± 4.3	25.3 ± 5.0	23.2 ± 5.4

SD = standard deviation. F/M = female/male.

* Adjustable stimulation group: 29 subjects completed 3 months of treatment, but analysis includes only the 28 who completed 3 months assessment of headache information in the electronic daily questionnaire.

### Changes in headache days, pain, and duration

**Figure 3.**
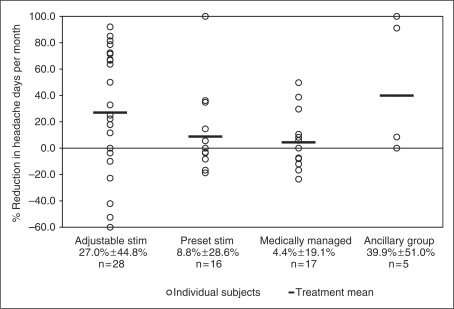
Percentage change in number of headache days. Adjustable stim = adjustable stimulation group. Preset stim = preset stimulation group.

The overall outcomes comparing baseline observations and three-month data are presented in Table 4. At three months, percent reduction in headache days per month was 27.0 ± 44.8% for AS, 8.8 ± 28.6% for PS, 4.4 ± 19.1% for MM and 39.9 ± 51.0% for the ancillary group. These percentages correspond to reductions in actual headache days per month of 6.7 ± 10.0 for AS, 1.5 ± 4.6 for PS, 1.0 ± 4.2 for MM and 9.1 ± 12.3 for the ancillary group. The reduction in overall pain intensity was 1.5 ± 1.6, 0.5 ± 1.3, 0.6 ± 1.0 and 1.9 ± 3.5 for AS, PS, MM and the ancillary group, respectively. The percent reduction in days with prolonged, severe headache per month was 24.4 ± 43.6% for AS, 10.3 ± 34.0% for PS, −1.2 ± 38.9% for MM and 33.5 ± 43.2% for the ancillary group. These percentages correspond to reduction in actual days per month of 5.1 ± 8.7 for AS, 2.2 ± 6.4 for PS, 0.8 ± 5.6 for MM and 7.7 ± 11.7 for the ancillary group. Figures 3 through 6 show the percentage change in number of headache days per month, change in overall pain intensity, percentage change in number of days with prolonged, severe headache and change in hours of headache per day across all four groups, respectively.

**Figure 4.**
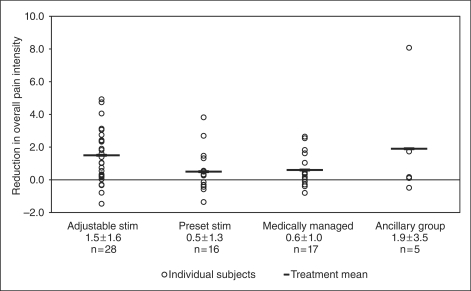
Change in overall pain intensity. Adjustable stim = adjustable stimulation group. Preset stim = preset stimulation group.

**Figure 5.**
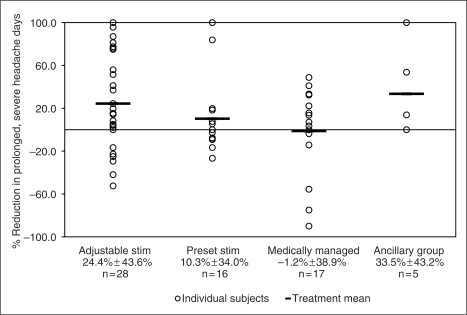
Percentage change in number of days with prolonged, severe headache. Adjustable stim = adjustable stimulation group. Preset stim = preset stimulation group.

**Figure 6.**
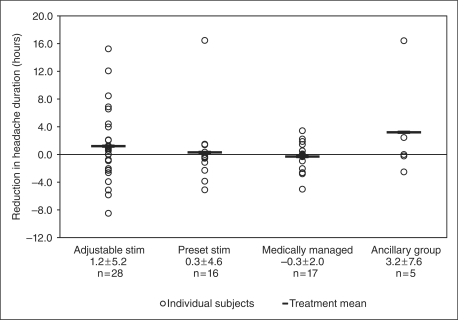
Change in hours of headache per day (averaged over all days). Adjustable stim = adjustable stimulation group. Preset stim = preset stimulation group.

**Table 4.** Percentage change in number of headache days

		Mean ± SD		
Treatment group	*N*	Baseline	3 months	Percentage change from baseline
Adjustable stimulation	28	22.4 ± 6.3	15.7 ± 10.0	27.0 ± 44.8
Preset stimulation	16	23.4 ± 5.1	21.9 ± 7.8	8.8 ± 28.6
Medically managed	17	23.7 ± 4.3	22.8 ± 6.3	4.4 ± 19.1
Ancillary	5	25.3 ± 5.0	16.3 ± 14.3	39.9 ± 51.0

SD = standard deviation.

For the majority of outcome measures (i.e. changes in headache days, pain and duration, including reduction in headache days, overall pain intensity, peak pain intensity, headache-free days, days with prolonged and severe headache and average headache duration), the exploratory analyses showed no statistically significant improvement over baseline when comparing the AS group with the control groups (PS and MM), although a numerical advantage appeared to be associated with the AS group. Because the number of subjects in the ancillary group was small, reliable comparisons could not be made.

### Responder rates

**Figure 7.**
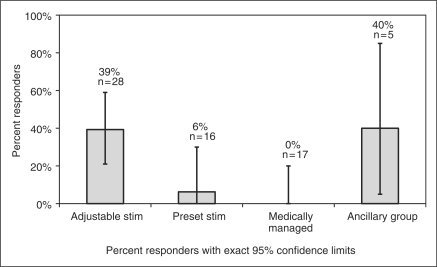
Responder rate by treatment group. Adjustable stim = adjustable stimulation group. Preset stim = preset stimulation group.

Figure 7 demonstrates responder rate across all four groups. Responder rate is the percentage of subjects who achieved a 50% or greater reduction in number of headache days per month or a three-point or greater reduction in average overall pain intensity compared with baseline. The responder rate in the AS group was 39%, compared with 6% in the PS group and 0% in the MM group. The differences between the AS and the control groups were significant in exploratory analyses.

### Disability and quality-of-life outcomes

The Profile of Moods States (POMS), MIDAS, SF-36, functional disability and subject satisfaction scores were also evaluated in this study. Exploratory analyses yielded significant differences between the AS and control groups in the measures reported here. The POMS was used to measure six areas of mood states: tension-anxiety, depression-dejection, anger-hostility, vigor-activity, fatigue-inertia and confusion-bewilderment. A lower score represents a decreased mood state. Reductions in POMS scores from baseline to three months were as follows: 8.7 ± 12.0 for AS, 1.6 ± 10.1 for MM and 0.4 ± 9.4 for PS. Sixty-six percent of subjects in the AS group and 25% of subjects in the MM group reported satisfaction with treatment at three months. Change from baseline in score on the functional disability scale was 0.3 ± 0.5 for the AS group and 0.0 ± 0.3 for the MM group. Change in acute medication use was 1.6 ± 7.6 in the AS group and −0.6 ± 5.0 in the MM group. Change in MIDAS average grade was 0.4 ± 0.8 for the AS group and 0.0 ± 0.0 for the MM group, and change in MIDAS headache pain score was 1.3 ± 1.8 for the AS group and 0.0 ± 0.9 for the MM group. Scores on the SF-36 Mental Health domain were 5.5 ± 9.7 and −1.5 ± 6.3 for the AS and MM groups, respectively.

For the majority of outcome measures of disability and quality of life, including the functional disability scale, MIDAS scores and SF-36, the exploratory analyses showed no significant improvement over baseline when comparing the AS group with the control groups (PS and/or MM), although a numerical advantage appeared to be associated with AS in most cases. Because the number of subjects in the ancillary group was small, reliable comparisons could not be made.

### Safety

#### Adverse device effects.

A total of 53 subjects underwent the implant procedure. Two subjects were intraoperative failures, leaving 51 subjects who were successfully implanted. Fifty-six ADEs occurred in 36 of the 51 subjects. Table 5 demonstrates the presence and frequency of ADEs in the 51 subjects. Three subjects experienced serious ADEs requiring hospitalization: implant site infection, lead migration and postoperative nausea. The most frequently reported ADE was lead migration, which occurred in 12 of 51 subjects (24%). There was no evidence of ADEs leading to long-term complications or potential nerve damage. There were no serious unanticipated ADEs reported or identified in this study.

**Table 5.** Presence and frequency of device-related adverse events

		Implanted subjects (*N* = 51)		
Category	Preferred term	No. of events	No. of subjects	Percentage of subjects
Surgery/anesthesia	Hypotension	1	1	2%
	Incision site complication	4	4	8%
	Post-procedural nausea	1	1	2%
	Post-procedural pain	1	1	2%
	Rash	1	1	2%
	Stitch abscess	1	1	2%
	Suture-related complication	1	1	2%
Programming	Migraine	1	1	2%
	Neck pain	1	1	2%
	Therapeutic product ineffective	6	6	12%
Neurostimulator	Neck pain	1	1	2%
	Sensation of pressure	1	1	2%
	Tenderness	1	1	2%
Neurostimulator pocket	Discomfort	1	1	2%
	Implant site hematoma	1	1	2%
	Implant site infection	3	2	4%
	Implant site irritation	1	1	2%
	Implant site pain	2	2	4%
Lead	High impedance	1	1	2%
	Lead fracture	1	1	2%
	Lead migration/dislodgment	12	12	24%
	Therapeutic product ineffective	2	2	4%
Lead/extension tract	Burning sensation	1	1	2%
	Extension migration/dislodgment	1	1	2%
	Implant site infection	8	7	14%
	Implant site inflammation	1	1	2%
Total		56	36	71%

#### Non–device-related adverse events.

Non–device-related adverse events involved principally worsening of migraine during the three-month testing period as compared with baseline. Nine percent of the AS group, 41% of the PS group and 24% of the MM group reported increased migraine. Adverse events related to medications were similar across treatment groups and ranged from 6% to 18%. Table 6 presents the non–device-related adverse events that were reported in more than one subject. The “total” row includes all events, including those reported in only one subject.

**Table 6.** Number of subjects with non–device-related adverse events, by study group*

	Adjustable stimulation (*N* = 33)		Preset stimulation (*N* = 17)		Medically managed (*N* = 17)		Ancillary (*N* = 8)	
Preferred term	No. of subjects	Percentage of subjects	No. of subjects	Percentage of subjects	No. of subjects	Percentage of subjects	No. of subjects	Percentage of subjects
Migraine	3	(9.1%)	7	(41.2%)	4	(23.5%)	0	(0.0%)
Drug toxicity	3	(9.1%)	2	(11.8%)	0	(0.0%)	1	(12.5%)
Headache	1	(3.0%)	2	(11.8%)	0	(0.0%)	1	(12.5%)
Adverse drug reaction	1	(3.0%)	1	(5.9%)	1	(5.9%)	0	(0.0%)
Sinusitis	0	(0.0%)	1	(5.9%)	1	(5.9%)	1	(12.5%)
Anxiety	1	(3.0%)	0	(0.0%)	1	(5.9%)	0	(0.0%)
Bronchitis	1	(3.0%)	1	(5.9%)	0	(0.0%)	0	(0.0%)
Depression	2	(6.1%)	0	(0.0%)	0	(0.0%)	0	(0.0%)
Dizziness	0	(0.0%)	1	(5.9%)	0	(0.0%)	1	(12.5%)
Fall	2	(6.1%)	0	(0.0%)	0	(0.0%)	0	(0.0%)
Fungal infection	0	(0.0%)	2	(11.8%)	0	(0.0%)	0	(0.0%)
Hypothyroidism	0	(0.0%)	2	(11.8%)	0	(0.0%)	0	(0.0%)
Lymphadenopathy	1	(3.0%)	1	(5.9%)	0	(0.0%)	0	(0.0%)
Sinus headache	1	(3.0%)	0	(0.0%)	1	(5.9%)	0	(0.0%)
Upper respiratory tract infection	0	(0.0%)	1	(5.9%)	1	(5.9%)	0	(0.0%)
Total^†^	17	(52%)	13	(76%)	9	(53%)	6	(75%)

* Adverse events reported in more than one subject.

† “Total” row includes all events, including those reported in only one subject.

## Discussion

The data from this feasibility study suggest ONS for medically intractable CM can be carried out relatively safely and is worthy of further study for this indication. Although the study was not prospectively powered for efficacy evaluation, the 39% responder rate in the AS group is comparable with response rates achieved with widely used preventive CM treatments, such as topiramate (16,17). Moreover, findings from the current study are consistent with recent work suggesting that the response to ONB may not predict treatment outcomes of ONS in primary headache (18–20). However, it is important to note that data from the current study and others regarding the predictive value of ONB must be interpreted with caution because of their small sample sizes. The value of response to ONB as a predictive factor remains to be determined, with the results suggesting this issue needs resolving in future studies.

The decision to employ ONS for the diagnosis of CM rather than for occipital neuralgia is based on the clinical phenotyping of patients responding to therapy (7). This is not to eliminate headache of cervical origin as a candidate for ONS; indeed, a retrospective review of cases responding to the procedure demonstrated a substantial cervicogenic cohort (21). Because CM is among the most refractory and costly to treat of the primary headache disorders and because chronic daily or near-daily headache affects up to 4% of the public (22), it was reasoned that if neurostimulation was effective and safe in treating this population, it would represent an important therapeutic contribution to quality of life for those who suffer from this illness and perhaps for long-term cost control as well.

Although the complete pathophysiology of CM remains unclear, a role for the trigeminocervical complex seems established (23). The intermingling of fibers from trigeminal afferents with those from cervical inputs, especially those of C2, underpins the phenotype of many primary headaches, including migraine (24). It is clear from experimental studies even in nonhuman primates (25) that second-order trigeminocervical afferents are involved in dura-vascular nociceptive transmission. Indeed, direct stimulation of C2 afferents can excite second-order trigeminal afferents (26). Direct evidence of this can be seen in patients with greater stimulation of the occipital nerves (27) and, indeed, from the distribution of pain, which ignores cutaneous innervation boundaries. Moreover, clinical experience whereby occipital nerve injections have been used in the management of both migraine (28,29) and cluster headache (30,31) reinforce the potential for treatment of these disorders by manipulation of the nerve. Functional imaging work has demonstrated changes in thalamic activation with ONS in CM (7), without change in the underlying brainstem activation (32), suggesting a neuromodulatory mechanism for this potential therapy.

This study indicates that the adverse events from the period of implantation to three months are not prohibitive to further exploration of therapy. The primary ADEs involved lead migration or dislodgement and incision-site complications. Lead migrations, although not generally defined as a serious adverse event, ultimately require a repeat invasive procedure and therefore should not be minimized in importance. There was no evidence of ADEs leading to long-term complications or potential nerve damage. Additional studies are currently reviewing the long-term effects of ONS. No unanticipated ADEs occurred in this study. Moreover, from this study we have gained sufficient technical information to move toward reducing lead migration in future studies, including considerations of appropriate strain-relief loops, anchors, implant locations, implant procedures and lead migration assessment techniques.

Even if the design and power of this study were sufficient to provide statistically reliable results, a responder rate of 39%, or any of the other positive findings reported here, might not seem compelling in support of neurostimulation. Nonetheless, from the perspective of an often treatment-refractory population of patients, such a finding could represent an important therapeutic signal for at least a subset of patients that justifies continued study in pursuit of ultimate therapeutic value. Patients with CM are often left without effective treatment, causing patients to lead lives that are painful and compromised. Patients with CM often travel from physician to physician and center to center, and are prescribed long lists of powerful medications, many in complex combinations that both compromise function and impose risk. Were a well-designed, controlled, and blinded study to demonstrate that even a small subset of patients with CM achieved reliable, substantial, prolonged benefit from ONS, we believe it would represent an important contribution to the care of these patients.

This study had several limitations. First, trial duration was short. A longer period of observation might reveal a different pattern of adverse events. Second, complete patient and investigator blinding was difficult to achieve. Although subjects and neurologists were blinded, implanters were necessarily unblinded for conduct of the study. In addition, maintaining subjects in a blinded mode is difficult in any study when stimulation can be perceived. The PS group did not have a device programmer, which also could have led to unblinding. Third, the preset stimulation might have, itself, had a therapeutic benefit, although this did not appear to be the case in the current study. Finally, the parameter of headache days could have used more sensitive definition, such as days with moderate or severe headache specified by not only intensity but also duration.

Many questions beyond basic efficacy remain unanswered for this therapy. Long-term safety of stimulation, durability of any positive benefit and technical and electronic reliability remain untested. Psychological and clinical eligibility for stimulation, the effect of medications on stimulation response (or lack thereof), interventional technique considerations and device and lead design factors have yet to be determined or fully explored.

There are many challenges to overcome before reliable conclusions on matters of efficacy and safety of ONS in medically intractable CM can be established. Developing a bona fide placebo group for surgical implant studies is particularly important—and particularly difficult. The perception of paresthesia may be required to obtain pain control, but technical issues make creation of this sensation in a placebo group a major challenge for the design of randomized controlled trials. Although the PS group represents a step to address this issue, more must be done to assure that the placebo stimulus is not itself therapeutically effective. In addition, better screening criteria are needed. However, because medically intractable CM is a frequent cause of disability and a therapeutic challenge in neurological practice, attempting to overcome these in future studies is a worthwhile pursuit.

## Conclusion

On the basis of the current findings and in light of previously published work, we believe further investigational pursuit to evaluate the efficacy and safety of ONS for medically intractable CM is justified. Further study would be enhanced by improved stimulator design, implanting technique and lead design and by a well-targeted, carefully selected study population, more robust endpoints, longer trial duration and improved blinding techniques. Reliable conclusions regarding efficacy cannot be established on the basis of this study alone. Nonetheless, the results of this feasibility study offer promise and should prompt further study of ONS in medically intractable CM.

## Authorship and disclosure

The study was designed by the authors and employees of Medtronic Neuromodulation. The full data set has been available to the authors, and data were analyzed by Medtronic. The lead authors (JRS, PJG) wrote the first draft of the manuscript. All content, interpretation, implications of results, discussion and conclusions were composed by the authors.

JRS has received consulting/advising compensation for activities with Merck, Ortho-McNeil-Janssen Pharmaceutical (OMP), Purdue Pharma, Neuralieve, Allergan, Medtronic, Pfizer and St. Jude Medical. He holds stock and/or stock options in Pozen. He receives research support from GlaxoSmithKline (GSK), Johnson & Johnson, Eli Lilly, Merck, St. Jude Medical, Map Pharma, Nupathe, Zogenix, Neura Axon and Boston Scientific. DWD has provided consulting services for the following companies within the past two years: Allergan, Addex, Alexza, Coherex, Eli Lilly, Endo, GSK, HS Lundbeck Kowa, MAP Pharmaceuticals (MAP), Medtronic, Merck, Minster, Nautilus, Neuralieve, Neuraxon, Novartis, NuPathe, Pfizer and Zogenix. DWD has received editorial honoraria from Wiley-Blackwell, the American College of Physicians, SAGE Publications and the *Neurologist,* and research support within the past two years from Medtronic, Advanced Neuromodulation Systems, St. Jude Medical, the National Institute of Neurological Disorders and Stroke and Mayo Clinic. SDS has consulted for Novartis and is on the speakers bureau of Endo Pharmaceuticals, GSK and Merck. SDS has served on advisory panels of Medical Corporation (AGA), Allergan, Capnia, Coherex, GSK, Lilly, MAP, Medtronic, Merck, NuPathe and Pfizer, and has received research support from AGA, Advanced Neuromodulation Systems, Allergan, Boston Scientific, Coherex, Endo Pharmaceuticals, GSK, Lilly, MAP, Medtronic, Merck, NuPathe and Valeant Pharmaceuticals. SM is an employee of Medtronic Neuromodulation and owns Medtronic stock through employment. MS is an employee of Medtronic Neuromodulation and owns Medtronic stock through employment. PJG has consulted for, and received honoraria from, ATI, Medtronic and Boston Scientific in the area of neuromodulatory treatment of headache.
